# Comparative Characterization of Oil Body Proteins from Hemp, Plum, and Jujube Seed and Their Application in Curcumin-Loaded Artificial Oleosomes

**DOI:** 10.3390/polym17101346

**Published:** 2025-05-15

**Authors:** Yuhan Cao, Qin Hu, Feng Xue

**Affiliations:** 1 School of Pharmacy, Nanjing University of Chinese Medicine, Nanjing 210023, China; caoyh6666@163.com (Y.C.); qinh1003@163.com (Q.H.); 2 Jiangsu Key Laboratory of Medicinal Substance and Utilization of Fresh Chinese Medicine, Nanjing University of Chinese Medicine, Nanjing 210023, China

**Keywords:** oil body proteins, artificial oleosomes, emulsifying properties, structural characterization, curcumin encapsulation, plum seed

## Abstract

The structural and functional characteristics of oil body proteins (OBPs) isolated from hemp, plum, and jujube seeds were systematically investigated, along with their potential application in constructing curcumin-loaded artificial oleosomes (AOs). OBPs were extracted through alkaline extraction coupled with ultrasonic disruption, followed by comprehensive physicochemical characterization using SDS-PAGE, FTIR spectroscopy, fluorescence spectroscopy, and evaluation of particle size, zeta potential, surface hydrophobicity, solubility, thermal stability, and emulsification properties. Plum seed-derived OBPs were found to demonstrate superior emulsifying capacity and solubility, which were attributed to distinctive structural features, including the following: an elevated random coil content (13%), enhanced surface hydrophobicity (21,781 A.U.), reduced particle size (103 nm), and higher zeta potential (−46 mV). These structural advantages were correlated with improved interfacial adsorption capacity and colloidal stability. When employed in AO fabrication, plum seed OBPs produced curcumin-loaded systems exhibiting maximum encapsulation efficiency (92%), minimal droplet size (5.99 μm), and optimal bio-accessibility (50%) compared to their hemp- and jujube-based counterparts. Furthermore, AOs utilizing plum seed OBPs displayed enhanced antioxidant activity and significantly improved stability. The collective findings establish plum seed OBPs as exceptional natural emulsifiers with strong potential for bioactive compound delivery applications.

## 1. Introduction

Plant-derived oil body proteins (OBPs) have garnered significant attention in recent years as natural emulsifiers for stabilizing lipid-based delivery systems in food and pharmaceutical applications [1]. These intrinsic structural proteins, primarily comprising oleosins, caleosins, and steroleosins, are anchored to oil bodies in seeds and play critical roles in maintaining lipid droplet stability through their amphipathic structure [2]. Unlike synthetic emulsifiers, OBPs offer biocompatibility, biodegradability, and functional versatility, aligning with the growing demand for clean-label ingredients. However, the emulsifying performance of OBPs varies substantially across plant sources due to differences in protein composition, structural conformation, and surface properties [3]. While OBPs from conventional oilseeds (e.g., soybean [4], rape [5], sunflower [6]) have been extensively studied, non-traditional sources such as hemp, plum, and jujube seeds remain underexplored despite their potential to yield OBPs with unique functional attributes. This knowledge gap limits the development of tailored emulsifiers for specialized applications, necessitating systematic comparisons of OBPs from diverse botanical origins.

Artificial oleosomes (AOs), reconstituted oil-in-water emulsions stabilized by OBPs, represent a promising strategy to enhance the delivery of lipophilic compounds [1]. These systems mimic natural oil bodies, leveraging the inherent emulsifying and stabilizing capacities of OBPs to protect encapsulated compounds from degradation while improving gastrointestinal release. Curcumin, a polyphenol with potent antioxidant [7], antimicrobial [8], antihyperlipidemic [9], and anti-inflammatory [10] properties, serves as an ideal model compound for AO-based delivery due to its hydrophobicity. However, the fundamental structure–function relationships remain poorly understood between (1) the native physicochemical characteristics of OBPs (including solubility, interfacial behavior, and structural conformation), (2) the supramolecular architecture of resulting AOs, and (3) their functional performance in terms of curcumin encapsulation efficiency, biological activity, and, ultimately, bioavailability. This knowledge gap significantly hinders the rational design of optimized delivery systems.

This study addresses these gaps by systematically investigating OBPs extracted from hemp, plum, and jujube seeds, with three objectives: (1) elucidate source-dependent variations in OBP structure (subunit composition, secondary/tertiary conformation, surface properties), (2) correlate structural features with emulsifying performance and colloidal stability, and (3) evaluate the efficacy of OBPs in fabricating curcumin-loaded AOs, focusing on encapsulation efficiency, antioxidant activity, and bio-accessibility. By integrating characterization techniques with functional assays, this work establishes structure–function relationships that inform the selection of optimal OBPs for bioactive delivery.

## 2. Materials and Methods

### 2.1. Materials

Hemp seeds were obtained from Shaanxi Chuliang Agricultural Technology Co., Ltd. (Bama, Guangxi, China). Plum and jujube seeds were sourced from Jiangsu Chengkai Traditional Chinese Medicine Co., Ltd. (Huaian, China). Curcumin (≥98% purity), medium-chain triglyceride, 2,2-diphenyl-1-picrylhydrazyl (DPPH), 2,2′-azino-bis (3-ethylbenzothiazoline-6-sulfonic acid) (ABTS), ammonium 8-phenyl-1-naphthalenesulfonate (ANS), pepsin (from porcine gastric mucosa, ≥15,000 U/g), trypsin (from porcine pancreas, ≥2500 U/mg), and bile salt (porcine origin) were procured from Shanghai Yuanye Biotechnology Co., Ltd. (Shanghai, China). Other reagents used in this study were purchased from Sinopharm Chemical Reagent Co., Ltd. (Shanghai, China). All the reagents used in this study were of analytical grade and were used as received.

### 2.2. Extraction of Oleosomes from Hemp, Plum, and Jujube Seed

Oleosomes were extracted from seeds using a modified alkaline extraction method adapted from a previous study [11]. Briefly, 100 g of seeds were soaked in 500 mL of 0.1 M NaHCO_3_ solution (pH 9.5) at 4 °C for 12 h. The hydrated seeds were then homogenized using a wall-breaking machine (Joyoung, Jinan, China) with intermittent pulsing (20 s on, 10 s off) for a total of 120 s to prevent overheating. The resulting slurry was filtered through double-layer cheesecloth, and the filtrate was centrifuged at 10,000× *g* (4 °C, 30 min). The upper oleosome-rich layer was carefully collected, resuspended in fresh NaHCO_3_ solution (0.1 M, pH 9.5), and recentrifuged under identical conditions. The final creamy phase containing purified oleosomes was collected for subsequent oil body protein (OBP) extraction.

### 2.3. Isolation of OBPs from Oleosomes

OBPs were extracted from oleosomes using a modified protocol based on a previous study with the following adjustments [3]. Briefly, the oleosomes were resuspended in an equal volume (1:1, *v*/*v*) of deionized water and subjected to ultrasonic disruption (400 W, 20 min total duration, 2 s on/2 s off pulse cycles; Scientz-IID, NingBo Scientz Biotechnology Co., Ltd., Ningbo, China) in an ice bath at 4 °C to facilitate protein release. The resulting suspension was then mixed with a 2:1 (*v*/*v*) solution of petroleum ether/ethanol (2.5:1, *v*/*v*) and vortexed thoroughly. Phase separation was achieved by centrifugation at 10,000× *g* for 20 min at 4 °C (Micro 21R, Thermo Fisher Scientific, Waltham, MA, USA). The OBPs-enriched intermediate layer was carefully collected, lyophilized (Labconco, Kansas City, MO, USA), and stored at 4 °C for subsequent analysis.

### 2.4. Physicochemical Properties of OBPs

#### 2.4.1. Analysis of Subunit Composition

The subunit composition of OBPs was analyzed by sodium dodecyl sulfate–polyacrylamide gel electrophoresis (SDS-PAGE) under denaturing conditions. Protein samples (4 mg/mL) were solubilized in loading buffer (Servicebio, Wuhan, China) containing 5% β-mercaptoethanol and denatured at 95 °C for 5 min. Electrophoresis was performed using a 12% precast polyacrylamide gel (Bio-Rad Laboratories, Hercules, CA, USA) at 200 V for 30 min in Tris–glycine–SDS running buffer (25 mM Tris, 192 mM glycine, 0.1% SDS, pH 8.3). Proteins were visualized by staining with Coomassie Brilliant Blue R-250 (Servicebio, Wuhan, China) for 1 h, followed by destaining in a methanol/acetic acid/water solution (45:10:45, *v*/*v*/*v*) until clear bands were observed.

#### 2.4.2. FTIR Spectroscopy for Secondary Structure Analysis

The protein secondary structure was analyzed by Fourier transform infrared (FTIR) spectroscopy. Lyophilized OBPs (2 mg) were mixed with potassium bromide (KBr) at a 1:100 (*w*/*w*) ratio and pressed into translucent pellets under 10-ton hydraulic pressure. FTIR spectra were acquired using a spectrometer (Lambda, Melbourne, Australia). Scans were performed from 4000 to 500 cm^−1^ at 4 cm^−1^ resolution with 32 accumulations under nitrogen purge to minimize atmospheric interference. The amide I region (1600–1700 cm^−1^) was baseline-corrected, subjected to second-derivative analysis, and deconvoluted using Gaussian curve fitting in OriginPro 2018 (OriginLab, Northampton, MA, USA) to quantify secondary structure components (α-helix, β-sheet, β-turns, and random coil).

#### 2.4.3. Intrinsic Fluorescence Spectroscopy for Tertiary Structure

The tertiary structure of OBPs was probed by intrinsic tryptophan fluorescence spectroscopy. Protein samples (2 mg/mL) were prepared in 10 mM phosphate buffer (pH 7.4) and centrifuged at 12,000× *g* for 10 min to remove aggregates. Fluorescence emission spectra (300–400 nm) were recorded using a spectrophotometer (Tecan, Männedorf, Switzerland) with an excitation wavelength of 290 nm.

#### 2.4.4. Particle Size and Zeta Potential of OBPs

The particle size and zeta potential of OBPs were measured by using a Zetasizer Nano ZS90 (Malvern Instruments Ltd., Malvern, UK). Samples were diluted to 0.2 mg/mL in ultrapure water (prefiltered through a 0.22 μm membrane) and sonicated for 10 min at 60 W.

#### 2.4.5. Microstructure of OBPs

The surface morphology of OBPs was characterized using scanning electron microscopy (SU8000, Hitachi High-Technologies, Tokyo, Japan). Protein samples were mounted on aluminum stubs using double-sided carbon conductive tape (Ted Pella, Inc., Redding, CA, USA) and sputter-coated with a 10 nm gold–palladium layer (60:40 Au/Pd) using an ion sputter coater (E-1045, Hitachi, Japan) under argon atmosphere to enhance surface conductivity. Imaging was performed at an accelerating voltage of 5 kV, using a secondary electron detector (Hitachi, Chiyoda, Japan) at 500× magnification. All samples were analyzed under high vacuum conditions (<5 × 10^−3^ Pa) to minimize charging effects.

#### 2.4.6. Surface Hydrophobicity of OBPs

The surface hydrophobicity of OBPs was quantified using ANS as a fluorescent probe, following the method of a previous study with modifications [12]. Protein samples were dissolved in 10 mM sodium phosphate buffer (pH 6.86) to obtain concentrations ranging from 0.03 to 1.0 mg/mL. A 1 mL aliquot of each protein solution was mixed with 10 μL of 0.05% (*w*/*v*) ANS solution (prepared in the same buffer) and incubated in the dark for 5 min at 25 °C to allow probe binding. Fluorescence measurements were performed using a microplate reader (Tecan, Männedorf, Switzerland) with the following parameters: excitation at 390 nm and emission at 470 nm. The relative surface hydrophobicity index (H_0_) was calculated as the initial slope of the linear regression curve plotting fluorescence intensity (arbitrary units) against protein concentration (mg/mL).

#### 2.4.7. Solubility of OBPs

The solubility of OBPs was quantitatively analyzed using the bicinchoninic acid (BCA) colorimetric method. Protein samples were initially dispersed in ultrapure water to obtain a 2 mg/mL stock solution. The dispersion was homogenized (10,000 rpm) for 2 min. Soluble fractions were subsequently separated by centrifugation (10,000× *g*, 20 min, 4 °C). For analysis, 20 μL aliquots of supernatant were combined with 200 μL of BCA working reagent (BCA Protein Assay Kit, Shanghai Yuanye Biotechnology Co., Ltd., Shanghai, China) in sterile 96-well flat-bottom polystyrene microplates. After thorough mixing (3 × 10 s vortex pulses at medium intensity), the reaction mixtures were incubated at 37 °C for exactly 30 min. Following incubation, plates were equilibrated to 25 °C for 5 min in ambient conditions. Absorbance was immediately measured at 562 nm using a microplate reader (Tecan, Männedorf, Switzerland). Protein solubility was calculated as the percentage ratio of soluble protein concentration in the supernatant to the total protein concentration.

#### 2.4.8. Thermal Stability of OBPs

The thermal transition behavior of OBPs was investigated using a differential scanning calorimeter (TA Instruments, New Castle, DE, USA). Protein samples (5 mg) were accurately weighed and hermetically sealed in aluminum crucibles with corresponding empty pans as references. Thermal scans were conducted from 25 °C to 200 °C at a constant heating rate of 10 °C min^−1^ under a continuous ultra-high purity nitrogen purge.

#### 2.4.9. Emulsifying Properties of OBPs

The emulsifying properties were evaluated according to a modified method based on previous studies [13]. Briefly, 80 mL of protein solution (2 mg/mL in 10 mM phosphate buffer, pH 7.0) was homogenized with 20 mL of hemp seed oil using a high-speed disperser (KINEMATICA AG, Lucerne, Switzerland) at 15,000 rpm for 2 min. The freshly prepared emulsion was immediately diluted 100-fold with 0.1% (*w*/*v*) sodium dodecyl sulfate solution to prevent droplet coalescence. Absorbance at 500 nm was measured at time zero (A_0_) and after 10 min (A_10_) using a UV-Vis spectrophotometer (UV-1900, Shimadzu Instruments Co., Ltd., Suzhou, Jiangsu, China) to calculate the emulsifying activity index (EAI) and emulsifying stability index (ESI).

### 2.5. Preparation of Artificial Oleosomes (AOs) Loaded with Curcumin

The fabrication of curcumin-encapsulated AOs was performed according to an established protocol with modifications [14]. Briefly, 1.5 g of OBPs and 1.0 g of phospholipids were dispersed in 77.5 g of ultrapure water under continuous magnetic stirring (500 rpm, 25 °C) for 30 min to ensure complete hydration. Subsequently, 20 g of medium-chain triglyceride containing 0.1% (*w*/*w*) curcumin was incorporated into the aqueous phase. The coarse emulsion was first prepared using a high-speed homogenizer (KINEMATICA AG, Switzerland) at 15,000 rpm for 5 min, followed by ultrasonication (400 W, 20 min, pulse mode: 2 s on/2 s off) with an ultrasonic probe (NingBo Scientz Biotechnology Co., Ltd., China) to refine the emulsion into AOs. The temperature was maintained at 4 °C throughout the process using an ice-water bath to prevent the thermal degradation of the curcumin.

### 2.6. Physicochemical Properties of AOs Loaded with Curcumin

#### 2.6.1. Encapsulation Efficiency

The encapsulation efficiency (EE) of curcumin in AOs was determined following an established protocol with modifications [15]. Briefly, freshly prepared AOs were mixed with ten volumes of ethanol to release unencapsulated curcumin. The mixture was vortexed (30 s) and subsequently centrifuged (10,000× *g*, 2 min, 4 °C). The supernatant containing free (non-encapsulated) curcumin was carefully collected, and its absorbance was measured at 425 nm with ethanol as the blank. A standard curve of curcumin in ethanol (1–10 μg/mL) was prepared for quantification.

#### 2.6.2. Particle Size and Zeta Potential

Prior to measurements, all samples were appropriately diluted (1:100 *v*/*v*) with deionized water to achieve optimal scattering intensity for measurements. Particle size distribution was determined using a laser diffraction particle size analyzer (LS 13320, Beckman Coulter Inc., Brea, CA, USA). The optical parameters were set with refractive indices of 1.330 for the dispersant (water) and 1.500 for the particles. Zeta potential measurements were performed using a Zetasizer Nano ZS90 system (Malvern Instruments Ltd., Malvern, UK).

#### 2.6.3. Microstructure

The structure of AOs was characterized using a fluorescence microscopy (CMS, Leica Microsystems, Wetzlar, Germany). For fluorescent labeling, 10 μL of fluorescein isothiocyanate (0.1 mg/mL in dimethyl sulfoxide) was mixed with 1 mL of AOs suspension (0.1% *w*/*v* in 10 mM phosphate buffer, pH 7.4) and incubated for 10 min at 25 °C in light-protected conditions [16]. Prior to imaging, 5 μL of the stained suspension was deposited on a microscope slide.

#### 2.6.4. Free Radical Scavenging Activity

The free radical scavenging activity was evaluated using DPPH and ABTS assays according to a previously established method [17]. Briefly, samples were diluted 50-fold with ethanol or (NH_4_)_2_S_2_O_8_ (2.45 mM), then mixed with a DPPH (0.2 mM) or ABTS (7 mM) solution (1:1 ratio). After 30 min incubation at 37 °C in the dark, absorbance was measured at 517 nm (DPPH) or 734 nm (ABTS).

#### 2.6.5. Bio-Accessibility of Curcumin

The bio-accessibility of curcumin was determined using an established in vitro digestion protocol [18]. AOs were first digested in simulated gastric fluid (2 mg/mL NaCl, 3.2 mg/mL pepsin, pH 2.0) at 37 °C for 2 h (2:1 *v*/*v* ratio), followed by intestinal digestion with simulated intestinal fluid (37.8 mg/mL bile salts, 0.33 mg/mL CaCl_2_, 2.8 mg/mL NaCl, 107.6 mg/mL trypsin, pH 7.0) at 37 °C for 2 h (2:3 *v*/*v* ratio). After centrifugation (10,000× *g*, 10 min, 4 °C), the digest separated into three layers: undigested lipids (top), curcumin-containing micellar phase (middle), and insoluble residues (bottom). Curcumin bio-accessibility (%) was calculated from absorbance measurements at 425 nm (micellar phase vs. total digest).

#### 2.6.6. Storage, Temperature, and Salt Stability

The stability of artificial oleosomes (AOs) was systematically evaluated through three complementary tests—storage stability, thermal stability, and ionic strength tolerance—following established protocols adapted from previous investigations [19]. AOs were stored at 4 °C for 7 days under dark conditions. Visual phase separation was monitored on day 7 using high-resolution imaging. Heat resistance was assessed through controlled thermal challenges simulating industrial processing conditions. AOs were subjected to two temperature–time regimens in a precision water bath: mild pasteurization (63 °C for 30 min) [20] and intensive thermal processing (95 °C for 10 min) [21]. Following heat treatment, samples were immediately cooled to 25 °C using an ice-water quenching protocol to terminate thermal effects. Salt-induced destabilization was investigated by supplementing AOs with NaCl to final concentrations of 50 mM and 250 mM. The salt-amended AOs were maintained at 25 °C for 12 h under static conditions prior to analysis.

### 2.7. Statistical Analysis

Triplicate experimental measurements were analyzed and expressed as mean values ± SD. Group comparisons were statistically evaluated through one-way ANOVA (SPSS v25.0, IBM Corp., Armonk, NY, USA), followed by Duncan’s post hoc testing (α = 0.05 threshold) to identify significant intergroup variations.

## 3. Results and Discussion

### 3.1. Structure of OBPs

#### 3.1.1. Subunits

**Figure 1:**
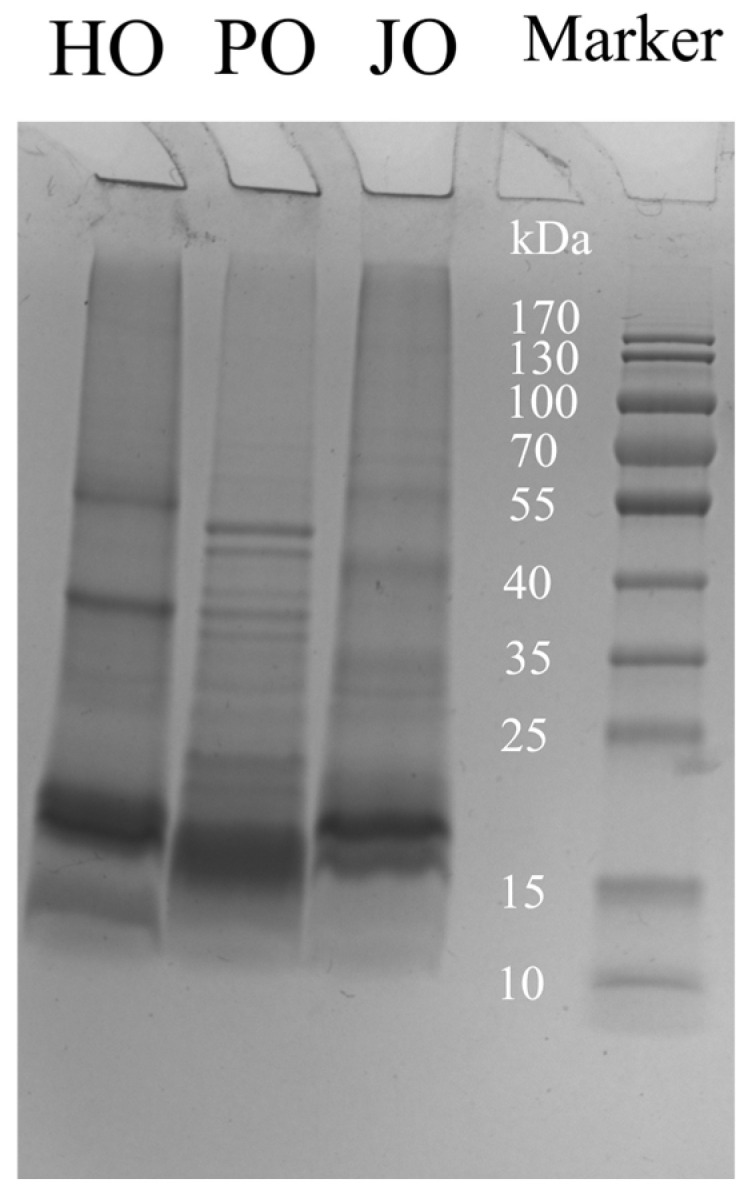
SDS-PAGE pattern of hemp seed oil body protein (HO), plum seed oil body protein (PO), and jujube seed oil body protein (JO).

As shown in Figure 1, the three oil body proteins (OBPs) were characterized through SDS-PAGE analysis, revealing predominant subunits within the 15–25 kDa range, which confirmed oleosins as the major protein components [2]. Caleosins were identified at 25–35 kDa, while high-molecular-weight proteins (>35 kDa) were categorized as steroleosins [2]. Among these OBPs, oleosins were found to possess superior emulsifying properties, which were ascribed to their characteristic amphipathic structure containing hydrophilic side chains and a hydrophobic central domain—a critical architectural feature for maintaining oleosome stability [22]. Molecular weight variations in OBPs across plant species have been documented in previous studies. For instance, soybean-derived OBPs were reported to exhibit molecular weights of 16, 24, and 28 kDa [23], while Camellia OBPs were observed at 14, 17, and 30 kDa [24]. In contrast, walnut and sesame OBPs were demonstrated to display broader molecular weight distributions spanning 12–67 kDa [25] and 15–41 kDa [26], respectively. These interspecies discrepancies were indicative of significant structural diversity among plant OBPs. Consequently, all three oleosome preparations were enriched with oleosins, thus explaining their enhanced emulsifying performance and suitability for artificial oleosome (AO) fabrication. Of particular interest, plum seed and jujube seed OBPs were observed to exhibit multiple electrophoretic bands within the 15–25 kDa region, suggesting the presence of distinct oleosin isoforms. This observation aligned with previous reports documenting three oleosin isoforms in soybean oleosomes [4], further supporting the evolutionary conservation of isoform diversity in oleosin proteins.

#### 3.1.2. FTIR Spectrum

**Figure 2:**
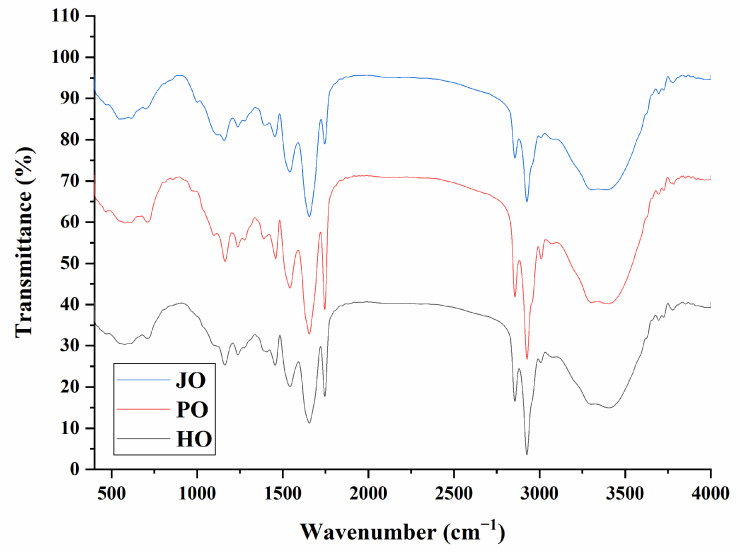
FTIR spectra of hemp seed oil body protein (HO), plum seed oil body protein (PO), and jujube seed oil body protein (JO).

Characteristic protein absorption bands were identified in two principal spectral regions through FTIR analysis (Figure 2). The amide A band (3300–3400 cm^−1^), which is associated with O-H/N-H stretching vibrations and hydrogen bonding networks [27], demonstrated pronounced intensity variations across the three OBP samples. Notably, the highest absorption intensity was observed in plum seed-derived proteins, suggesting more extensive intermolecular hydrogen bonding relative to other botanical sources [28]. Similar analytical approaches had been previously employed in soybean OBP investigations, where the amide A band was effectively utilized to evaluate hydrogen bonding interaction strength [23,29]. In the amide I region (1600–1700 cm^−1^), known to reflect protein secondary structure composition [30], distinct spectral profiles were recorded. These spectral variations were indicative of source-dependent differences in secondary structural organization. Specifically, the characteristic absorption patterns were found to vary significantly among the hemp, plum, and jujube OBPs, reflecting inherent variations in α-helix/β-sheet ratios and random coil configurations. The observed heterogeneity in secondary structure elements was consistent with previous reports documenting species-specific structural adaptations in OBPs [3].

#### 3.1.3. Secondary Structures

**Figure 3:**
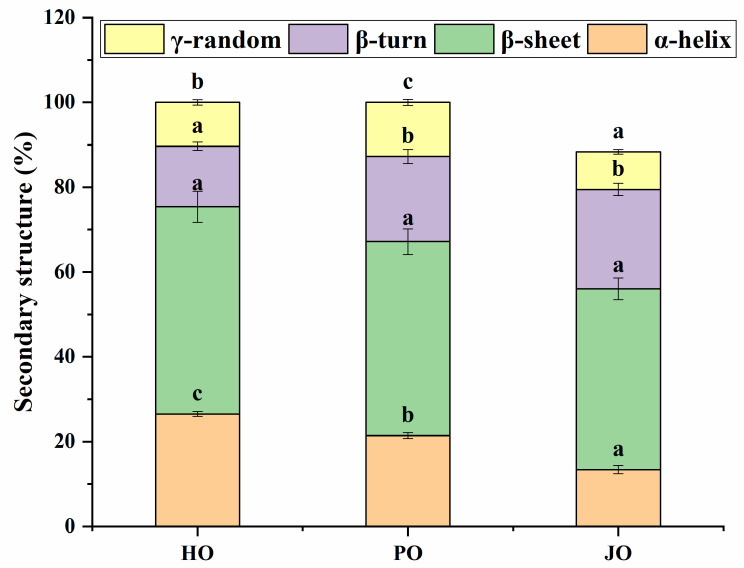
Secondary structures of hemp seed oil body protein (HO), plum seed oil body protein (PO), and jujube seed oil body protein (JO). Results with different letters within the same pattern are significantly different (*p* < 0.05).

As demonstrated in Figure 3, β-sheet structures were identified as the predominant secondary structural element across all three OBP variants. This structural prevalence was mechanistically significant given that β-sheet conformations have been recognized to stabilize internal protein architecture—a characteristic that has been strongly correlated with the distinctive hydrophobic core organization of OBPs [31]. These observations provided compelling evidence that the formation of well-defined hydrophobic domains constituted a fundamental structural prerequisite for effective OBP-mediated emulsification. The findings were corroborated by previous investigations demonstrating β-sheet dominance in OBPs from soybean [29], hemp seed, and cucumber seed [3]. Comparative analysis of secondary structures revealed significant variations in random coil composition among the OBP variants (Figure 3). From a functional perspective, these random coil segments were found to serve dual roles: (1) modulation of protein surface hydrophobicity [3], and (2) regulation of interfacial adsorption film stability at oil–water interfaces [32]. Notably, plum seed-derived OBPs were shown to possess significantly higher random coil content compared to other variants (*p* < 0.05). This structural distinction was interpreted as an indicator of enhanced emulsification capacity. Previous investigations of plum seed proteins had similarly demonstrated that increased random coil proportions in secondary structures could substantially improve emulsifying performance [33]. The superior interfacial activity observed in high-random-coil variants was found to align with established structure–function paradigms for plant proteins, where elevated conformational flexibility (as evidenced by increased random coil percentages) has been shown to facilitate more efficient adsorption at oil–water interfaces [34].

#### 3.1.4. Intrinsic Fluorescence Emission Spectra

**Figure 4:**
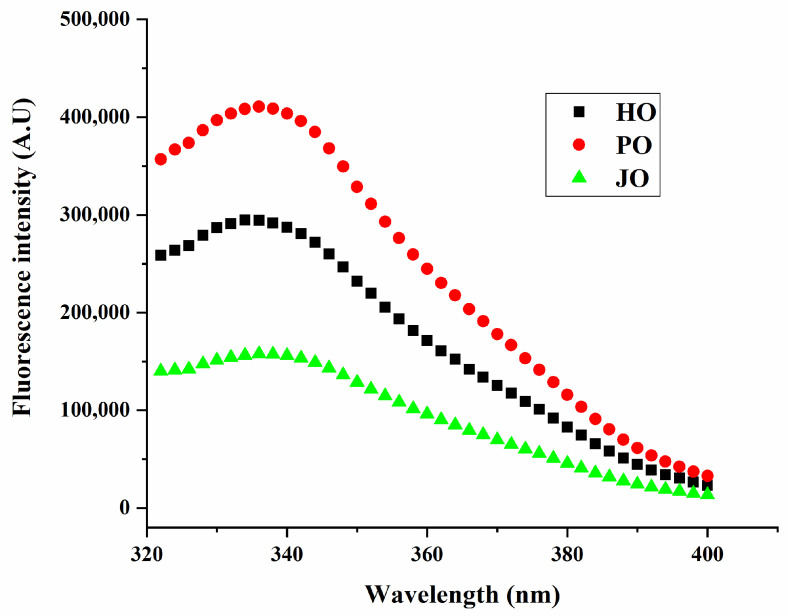
Fluorescence spectra of hemp seed oil body protein (HO), plum seed oil body protein (PO), and jujube seed oil body protein (JO).

Distinct structural properties of the three OBPs were characterized through fluorescence spectroscopic analysis, with plum seed-derived variants being observed to exhibit significantly enhanced emission intensity at 340 nm relative to hemp- and jujube-derived counterparts (Figure 4). The fluorescence emission profiles were interpreted as sensitive indicators of tryptophan residue microenvironments, where solvent-exposed tryptophan residues were subjected to dynamic quenching through collisional interactions with polar water molecules [35]. Tryptophan moieties in plum seed OBPs were found to be predominantly localized within hydrophobic regions, indicative of greater surface hydrophobicity compared to other OBP variants. Given that hydrophobic domains are typically sequestered within protein interiors, this finding suggested that plum seed OBPs might adopt a more relaxed tertiary conformation. This phenomenon had been previously documented in soybean OBPs, where a positive correlation between intrinsic fluorescence intensity and protein surface hydrophobicity was established [29]. The observed structural characteristics were found to be consistent with secondary structure analysis results, wherein plum seed OBPs were demonstrated to possess a characteristically elevated random coil content. This structural configuration was considered particularly advantageous for emulsification processes, as increased conformational flexibility has been shown to enhance interfacial adsorption efficiency at lipid–aqueous interfaces [33]. The collective evidence from fluorescence spectroscopy and secondary structure analysis confirmed that the superior emulsifying performance of plum seed OBPs originated from their unique structural attributes combining enhanced surface hydrophobicity with elevated structural flexibility.

### 3.2. Physicochemical Properties of OBPs

#### 3.2.1. Particle Size and Zeta Potential

**Figure 5:**
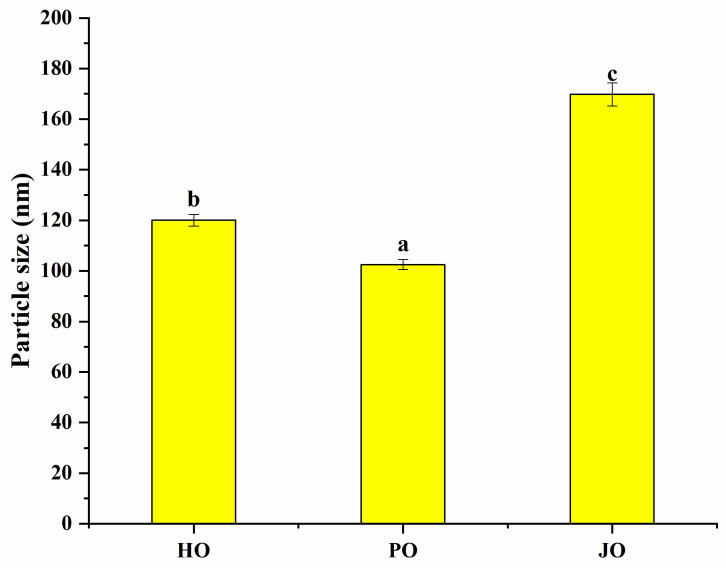
Particle size of hemp seed oil body protein (HO), plum seed oil body protein (PO), and jujube seed oil body protein (JO). Results with different letters within the same pattern are significantly different (*p* < 0.05).

As demonstrated in Figure 5, all OBP variants were observed to form nanoaggregates with diameters below 180 nm. The measured particle dimensions were found to be significantly smaller than those reported for soybean OBPs, which typically form aggregates of approximately 500 nm under neutral conditions [29]. Similarly, rapeseed-derived OBPs have been documented to generate aggregates smaller than 450 nm in aqueous environments [36]. This size confinement phenomenon was primarily attributed to the intrinsic amphiphilic nature of oleosins, where spontaneous self-assembly into nanoscale structures was driven by hydrophobic domain interactions in aqueous media [37]. Of particular significance, plum seed-derived OBPs were shown to exhibit the smallest particle dimensions among the three variants, indicative of superior colloidal characteristics. The reduced particle size was postulated to confer dual functional advantages: (1) enhanced hydration capacity through an increased surface area-to-volume ratio, and (2) improved adsorption kinetics at oil–water interfaces due to diminished diffusion limitations [38]. These observations were consistent with previous investigations of rapeseed oleosins, where 200 nm aggregates were formed in aqueous solutions [36]. The size-dependent colloidal properties were mechanistically linked to the structural features identified through spectroscopic analyses. Specifically, the combination of elevated surface hydrophobicity and increased conformational flexibility was considered instrumental in facilitating the formation of smaller, more stable nanoaggregates.

**Figure 6:**
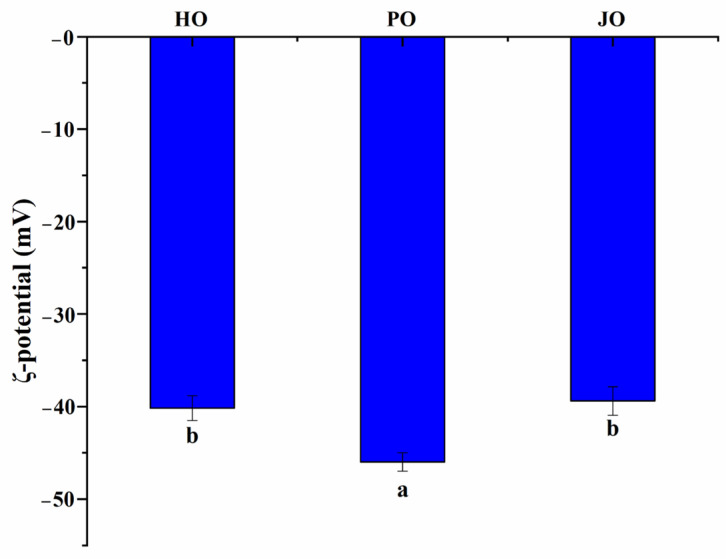
Zeta potentials of hemp seed oil body protein (HO), plum seed oil body protein (PO), and jujube seed oil body protein (JO). Results with different letters within the same pattern are significantly different (*p* < 0.05).

Zeta potential measurements were conducted to characterize the three OBP variants (Figure 6). All samples were found to possess substantial negative surface charges, generating sufficient electrostatic repulsion forces to prevent macroscopic aggregation. The surface charge characteristics of these OBPs were determined to be comparable to those documented for cucumber seed and perilla seed OBPs [3]. Comparative analysis revealed that a significantly higher surface charge density was exhibited by these proteins relative to soybean-derived OBPs, which have been reported to typically display ζ-potential values of approximately −20 mV [29]. Among the three variants, plum seed OBPs were observed to demonstrate the highest net surface charge (−46 mV), which was mechanistically linked to three critical factors: (1) enhanced interparticle repulsive forces, (2) an expanded conformational state enabling the exposure of internal charged groups, and (3) a minimized hydrodynamic diameter consistent with charge-stabilized colloidal systems. These structural–electrostatic characteristics were found to align with previous reports establishing that elevated surface charge density effectively prevented OBP flocculation through enhanced colloidal stabilization [39]. The observed surface charge properties were further correlated with prior analytical findings, particularly the increased random coil content. This synergistic structural configuration was considered to promote charge-mediated stabilization, thereby explaining the superior functional performance of plum seed OBPs in artificial oleosome fabrication.

#### 3.2.2. Microstructure

**Figure 7:**
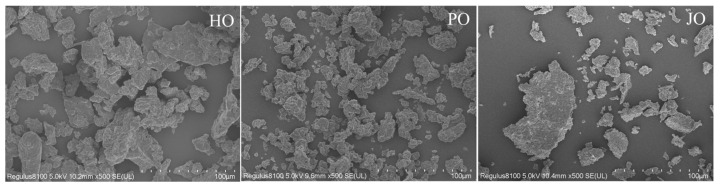
Microstructure of hemp seed oil body protein (HO), plum seed oil body protein (PO), and jujube seed oil body protein (JO).

Distinct aggregation morphologies were observed among the three OBP variants through scanning electron microscopy (SEM) analysis (Figure 7). Plum seed-derived OBPs were found to form significantly smaller aggregates compared to their hemp and jujube seed counterparts. Hemp seed OBPs were observed to exhibit intermediate-sized aggregates, while jujube seed OBPs demonstrated the most pronounced aggregation behavior, characterized by micrometer-scale cluster formation. During the lyophilization process, aggregates were typically formed through intermolecular interactions dominated by hydrophobic forces and hydrogen bonding networks [40]. The reduced aggregate dimensions observed in plum seed OBPs were mechanistically correlated with their elevated surface charge density (−46 mV), as quantified in zeta potential measurements (Figure 6). This observation was consistent with the colloidal stabilization mechanism described in previous sections, where stronger electrostatic repulsion forces were shown to effectively counteract aggregation tendencies.

#### 3.2.3. Surface Hydrophobicity

**Figure 8:**
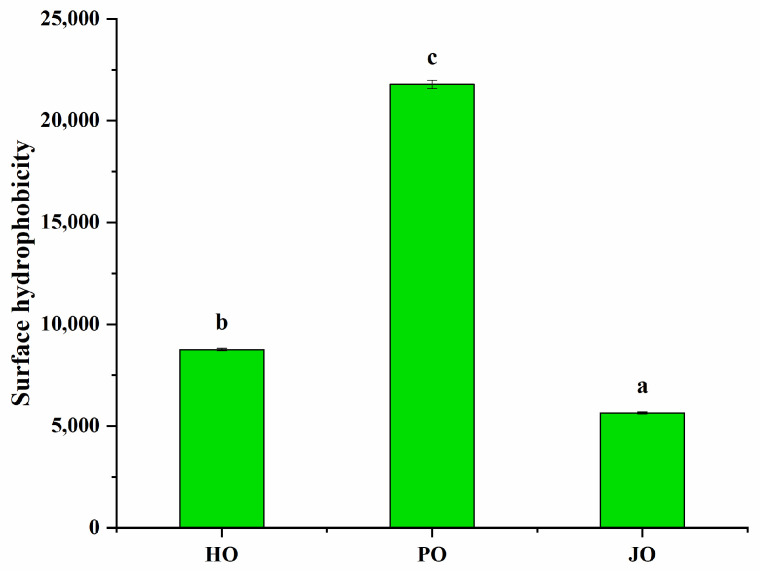
Surface hydrophobicity of hemp seed oil body protein (HO), plum seed oil body protein (PO), and jujube seed oil body protein (JO). Results with different letters within the same pattern are significantly different (*p* < 0.05).

Statistically significant differences in surface hydrophobicity were observed among the three OBP variants (*p* < 0.05), as demonstrated in Figure 8. Plum seed-derived OBPs were observed to demonstrate markedly higher surface hydrophobicity compared to hemp- and jujube-derived counterparts. This phenomenon was mechanistically attributed to two interconnected structural features: (1) A characteristically relaxed structural conformation, as evidenced by secondary structure analysis and tertiary structure evaluation, which collectively facilitated enhanced exposure of internal hydrophobic domains [41]. (2) Significantly reduced particle dimensions, consistent with established colloidal principles, where larger protein aggregates were shown to exhibit progressive surface hydrophobicity reduction due to steric masking effects [42]. This inverse relationship between particle size and surface hydrophobicity had been previously documented in soybean OBPs [29], confirming the universal applicability of this colloidal behavior across plant species.

#### 3.2.4. Solubility

**Figure 9:**
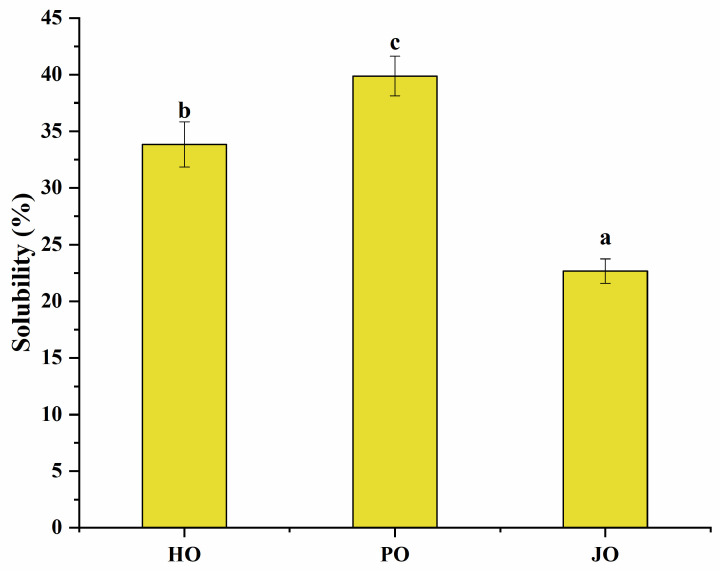
Solubility of hemp seed oil body protein (HO), plum seed oil body protein (PO), and jujube seed oil body protein (JO). Results with different letters within the same pattern are significantly different (*p* < 0.05).

Significant solubility differences were observed among the three OBP variants (*p* < 0.05), as shown in Figure 9. All tested OBPs were found to exhibit solubility values exceeding 20%, representing substantially higher water dispersibility compared to *Camellia oleifera* seed-derived OBPs, which demonstrated approximately 13% solubility [43]. Plum seed-derived OBPs were observed to display remarkably high solubility despite their elevated surface hydrophobicity—a phenomenon that appeared to contradict the well-established inverse correlation between hydrophobicity and solubility typically observed in plant proteins [44]. This apparent contradiction was explained through two synergistic mechanisms: (1) The nanoscale dimensions of plum seed OBPs were shown to enhance water interaction efficiency through increased surface area-to-volume ratios [45]. (2) The optimized surface hydrophobicity was found to promote the formation of stable soluble aggregates, where balanced hydrophobic interactions maintained colloidal stability while preventing macroscopic precipitation [40]. Therefore, the unique structural configuration of plum seed OBPs—combining high surface charge density (Figure 6), reduced particle dimensions (Figure 5), and elevated random coil content (Figure 3)—was identified as the critical factor enabling the simultaneous achievement of high hydrophobicity and exceptional solubility.

#### 3.2.5. Thermal Stability

**Figure 10:**
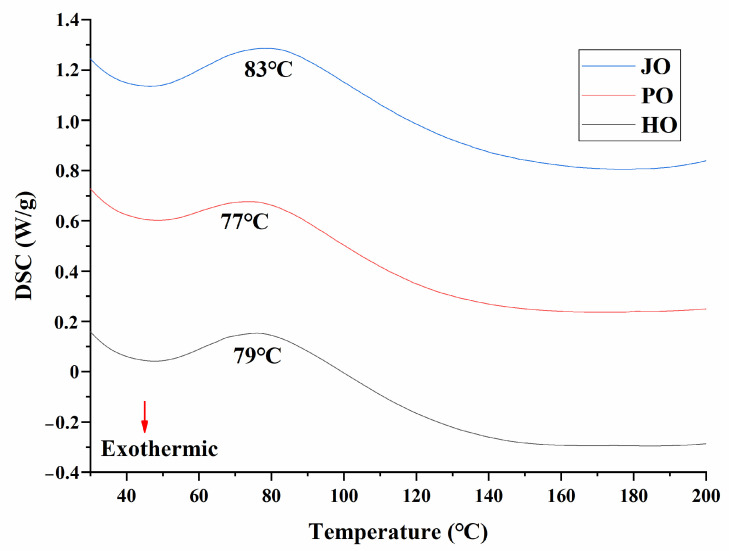
DSC curve of hemp seed oil body protein (HO), plum seed oil body protein (PO), and jujube seed oil body protein (JO).

Thermal denaturation characteristics were investigated through differential scanning calorimetry (DSC), with representative thermograms presented in Figure 10. All three OBP variants were observed to exhibit characteristic endothermic peaks within the 60–100 °C temperature range, corresponding to protein denaturation transitions. The thermal degradation temperature of plum seed proteins had been previously documented at approximately 74 °C [33], which aligned closely with the current experimental observations. Notably, plum seed-derived OBPs were found to demonstrate reduced thermal stability compared to other variants, as evidenced by a lower denaturation temperature. This diminished thermal resistance was mechanistically attributed to two structural factors: (1) an intrinsically relaxed structural conformation, as confirmed through secondary structure analysis and tertiary structure evaluation; (2) significantly reduced particle dimensions, which were shown to increase specific surface area compared to jujube and hemp seed OBPs, thereby enhancing heat transfer efficiency during thermal processing. The inverse relationship between structural compactness and thermal stability was consistent with previous reports on plant protein denaturation mechanisms [28].

#### 3.2.6. Emulsifying Properties

**Figure 11:**
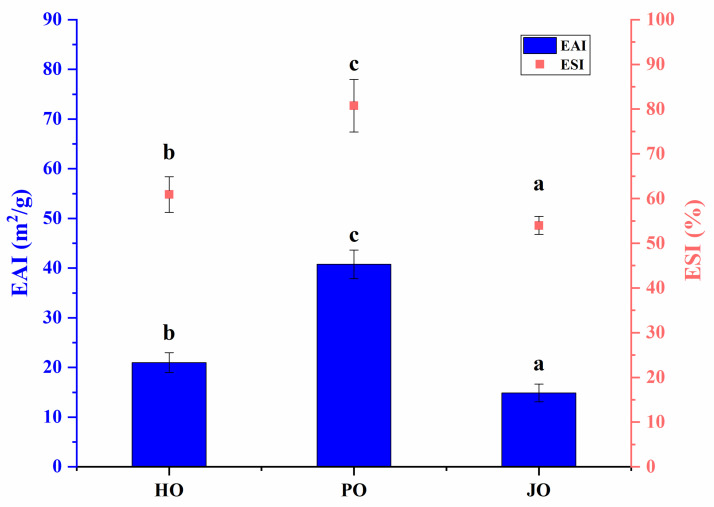
Emulsifying properties of hemp seed oil body protein (HO), plum seed oil body protein (PO), and jujube seed oil body protein (JO). Results with different letters within the same pattern are significantly different (*p* < 0.05).

As shown in Figure 11, statistically significant differences in emulsifying properties were observed among the three OBPs (*p* < 0.05). Superior emulsifying activity index (EAI) and emulsifying stability index (ESI) values were recorded for plum seed-derived OBPs compared to other variants. The emulsification characteristics of these OBPs were found to be comparable to those reported for soybean-derived OBPs, which demonstrated EAI values of 37 m^2^/g in previous studies [46]. The enhanced emulsifying properties of plum seed-derived OBPs were postulated to result from their distinct structural and physicochemical characteristics. These included unique molecular configurations, differential solubility profiles, optimized surface hydrophobicity, specific surface charge distributions, and reduced particle dimensions. Previous investigations have established that accelerated adsorption at oil–water interfaces can be induced by elevated surface hydrophobicity [47]. Improved diffusion kinetics have been associated with more flexible tertiary structures, diminished particle sizes, and enhanced solubility [48]. The formation of cohesive interfacial films has been linked to robust intermolecular interactions, particularly through hydrogen bonding and hydrophobic forces [3]. Furthermore, superior electrostatic stabilization against droplet coalescence has been correlated with increased surface charge density [49]. These findings align with earlier research on plant-derived proteins. Structural modifications characterized by protein unfolding, particle size reduction, and charge density augmentation were demonstrated to significantly improve emulsification capacity in hemp seed proteins [12]. Parallel observations were reported for plum seed proteins, where enhanced emulsification performance was directly associated with elevated surface hydrophobicity and improved solubility characteristics [33].

### 3.3. Physicochemical Properties of AOs Loaded with Curcumin

#### 3.3.1. Appearance

**Figure 12:**
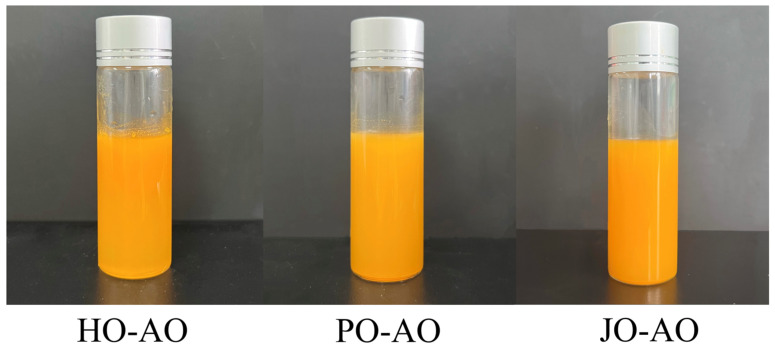
Appearance of artificial oleosomes (AOs) loaded with curcumin. HO-AO: artificial oleosomes stabilized by hemp seed oil body protein; PO-AO: artificial oleosomes stabilized by plum seed oil body protein (PO) and jujube seed oil body protein; JO-AO: artificial oleosomes stabilized by jujube seed oil body protein.

As illustrated in Figure 12, the stable dispersion of curcumin was observed in all three artificial oleosome (AO) systems, with no visible precipitation or phase separation detected, demonstrating their efficacy as delivery vehicles. These findings were considered to validate emulsion-based systems as an effective strategy for improving curcumin solubility [50,51].

#### 3.3.2. Encapsulation Efficiency

**Figure 13:**
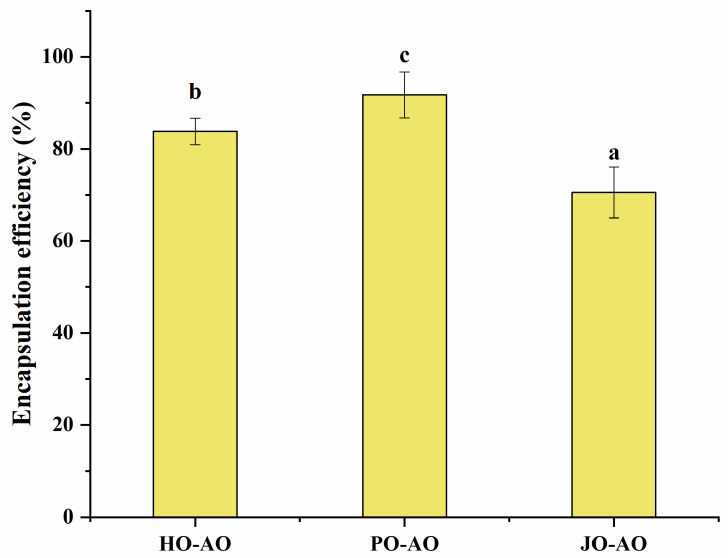
Encapsulation efficiency of artificial oleosomes (AOs) loaded with curcumin. HO-AO: artificial oleosomes stabilized by hemp seed oil body protein; PO-AO: artificial oleosomes stabilized by plum seed oil body protein (PO) and jujube seed oil body protein; JO-AO: artificial oleosomes stabilized by jujube seed oil body protein. Results with different letters within the same pattern are significantly different (*p* < 0.05).

Significant variations in curcumin encapsulation efficiency (EE) were observed among OBP variants in artificial oleosomes (*p* < 0.05, Figure 13). The highest EE value was recorded for plum seed-derived oleosomes, a phenomenon strongly correlated with their elevated surface hydrophobicity. Enhanced hydrophobic interactions between the OBP variants and the phenolic rings of curcumin were hypothesized to minimize curcumin leakage [52]. Notably, plum seed OBP-based AOs achieved an exceptional EE of 92%, which was comparable to or superior to natural polymer-based emulsion systems reported in the literature. Specifically, this performance paralleled chickpea protein/pectin emulsions (89%) [53], chitosan/starch emulsions (92%) [54], and starch/palmitic acid/β-lactoglobulin complex emulsions (93%) [55], while exceeding whey protein/fucoidan emulsions (76%) [56].

#### 3.3.3. Particle Size and Zeta Potential

**Figure 14:**
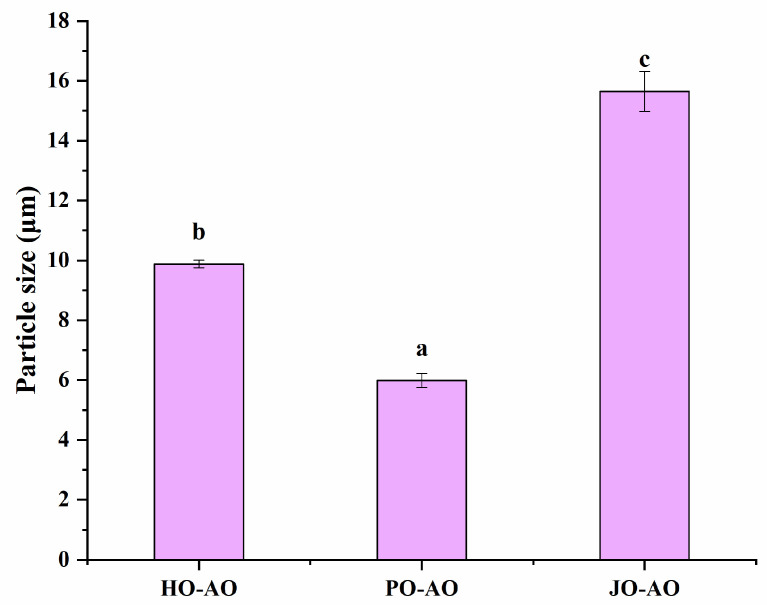
Particle size of artificial oleosomes (AOs) loaded with curcumin. HO-AO: artificial oleosomes stabilized by hemp seed oil body protein; PO-AO: artificial oleosomes stabilized by plum seed oil body protein (PO) and jujube seed oil body protein; JO-AO: artificial oleosomes stabilized by jujube seed oil body protein. Results with different letters within the same pattern are significantly different (*p* < 0.05).

As demonstrated in Figure 14, particle sizes of curcumin-loaded AOs were significantly influenced by OBP variants (*p* < 0.05), with plum seed-derived oleosomes exhibiting the smallest mean diameter. This size reduction was mechanistically attributed to two factors: (1) superior emulsification capacity, which facilitated the formation of dense interfacial films to inhibit droplet coalescence, and (2) increased surface hydrophobicity, which promoted rapid adsorption at oil–water interfaces during homogenization. These observations aligned with established structure–function relationships, wherein proteins with high emulsifying capacity [57] and surface hydrophobicity [58] have been consistently associated with smaller emulsion particle sizes. Furthermore, the particle size distribution of plum seed OBP-based AOs was comparable to that of natural oleosomes, closely resembling those of hemp seed oleosomes (2 μm) [59] and rapeseed oleosomes (1 μm) [60], though larger than those of soybean oleosomes (376 nm) [61] and peanut oleosomes (600 nm) [62].

**Figure 15:**
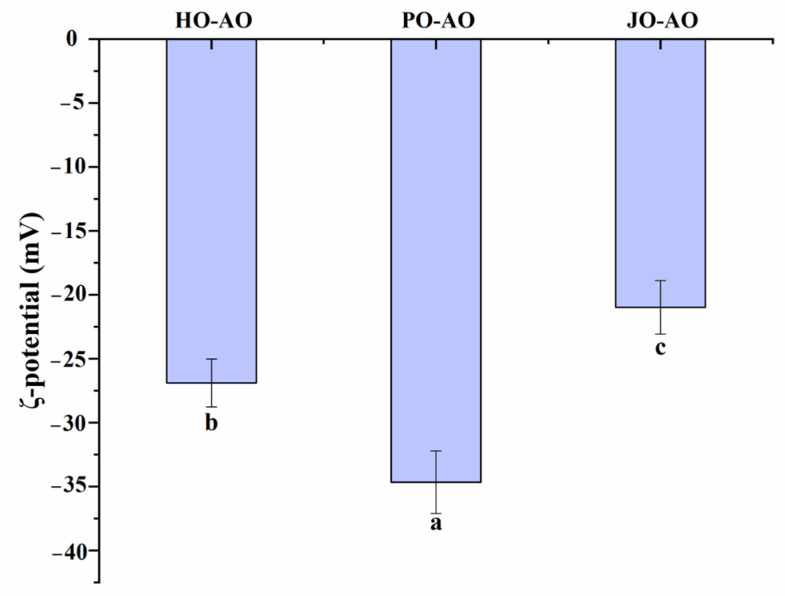
Zeta potential of artificial oleosomes (AOs) loaded with curcumin. HO-AO: artificial oleosomes stabilized by hemp seed oil body protein; PO-AO: artificial oleosomes stabilized by plum seed oil body protein (PO) and jujube seed oil body protein; JO-AO: artificial oleosomes stabilized by jujube seed oil body protein. Results with different letters within the same pattern are significantly different (*p* < 0.05).

As shown in Figure 15, significant variations in surface charge density were detected among curcumin-loaded AOs depending on OBP sources (*p* < 0.05). Plum seed-derived oleosomes displayed the lowest zeta potential, a feature attributed to the intrinsically higher net charge of plum seed OBPs. A positive correlation between oleosin net charge and emulsion surface charge density has been well-documented in previous studies [39]. Additionally, the ζ-potential of plum seed OBP-based AOs (−35 mV) matched values reported for natural oleosomes, including peanut (−35 mV) [62], soybean (−29 mV) [61], and rapeseed (−30 mV) [60] variants.

#### 3.3.4. Microstructure

**Figure 16:**
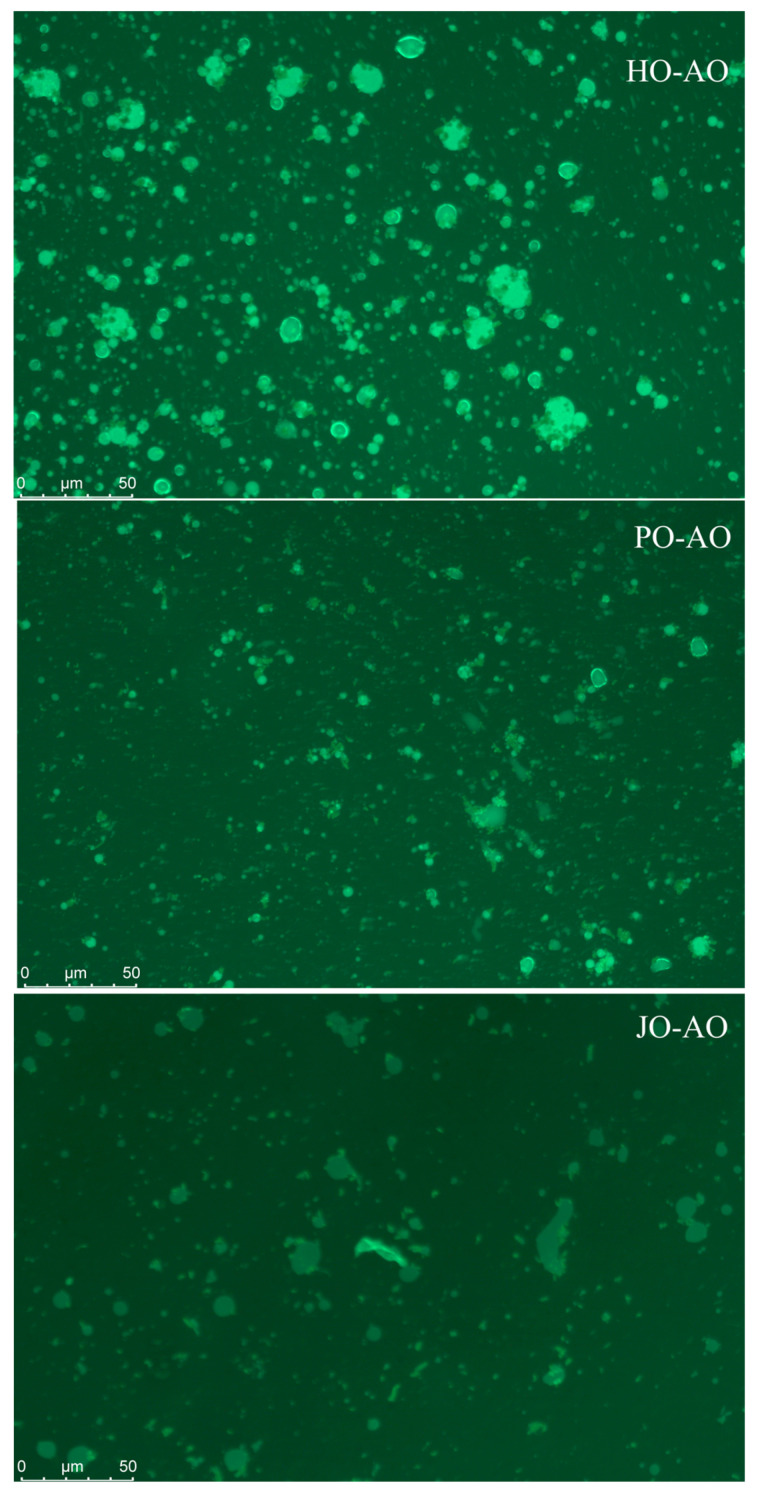
Microstructure of artificial oleosomes (AOs) loaded with curcumin. HO-AO: artificial oleosomes stabilized by hemp seed oil body protein; PO-AO: artificial oleosomes stabilized by plum seed oil body protein (PO) and jujube seed oil body protein; JO-AO: artificial oleosomes stabilized by jujube seed oil body protein.

As demonstrated in Figure 16, plum seed-derived AOs were observed to form significantly smaller and more uniformly distributed oil droplets compared to hemp- and jujube-derived variants, which exhibited microscopically visible large aggregates. These observations were found to align with particle size distribution analyses, suggesting that the superior colloidal stability of plum seed AOs could be attributed to their intrinsically higher surface net charge (−46 mV), generating enhanced electrostatic repulsion forces to prevent droplet coalescence. This stabilization mechanism was consistent with the established literature demonstrating that elevated surface charge density reduces flocculation through strengthened inter-droplet repulsion [63].

#### 3.3.5. Free Radical Scavenging Activity

**Figure 17:**
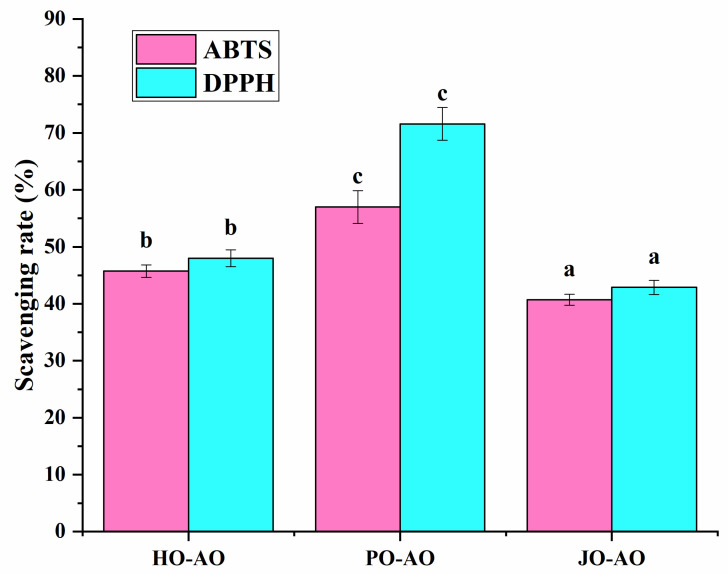
ABTS and DPPH scavenging activity of artificial oleosomes (AOs) loaded with curcumin. HO-AO: artificial oleosomes stabilized by hemp seed oil body protein; PO-AO: artificial oleosomes stabilized by plum seed oil body protein (PO) and jujube seed oil body protein; JO-AO: artificial oleosomes stabilized by jujube seed oil body protein. Results with different letters within the same pattern are significantly different (*p* < 0.05).

All AO variants were shown to exhibit free radical scavenging capacity (Figure 17), which was attributed to two synergistic factors: (1) encapsulated curcumin with inherent antioxidant properties, and (2) intrinsic radical-neutralizing activity of the OBPs themselves. This antioxidant mechanism had been previously documented in soybean OBPs, where antioxidant amino acid residues were identified as the primary contributors to radical scavenging [23]. Notably, plum seed-derived AOs demonstrated greater radical scavenging efficiency compared to other variants, a phenomenon mechanistically linked to their reduced particle dimensions. The smaller droplet size was postulated to enhance antioxidant performance through two pathways: (1) accelerated dispersion kinetics in aqueous assay systems, and (2) an increased interfacial contact area for radical interaction [64].

#### 3.3.6. Bio-Accessibility of Curcumin

**Figure 18:**
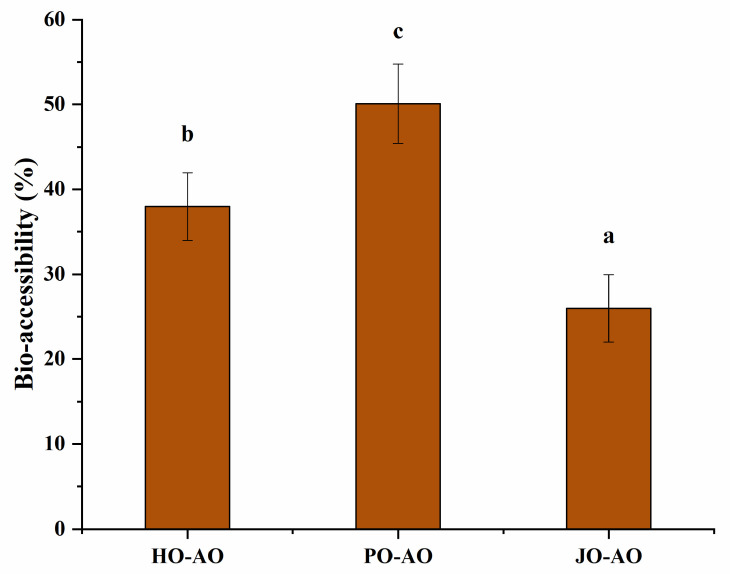
Bio-accessibility (C) of artificial oleosomes (AOs) loaded with curcumin. HO-AO: artificial oleosomes stabilized by hemp seed oil body protein; PO-AO: artificial oleosomes stabilized by plum seed oil body protein (PO) and jujube seed oil body protein; JO-AO: artificial oleosomes stabilized by jujube seed oil body protein. Results with different letters within the same pattern are significantly different (*p* < 0.05).

As demonstrated in Figure 18, the plum seed-derived AOs exhibited significantly enhanced curcumin bioavailability due to two synergistic mechanisms: (1) The smaller particle size was shown to promote bile salt adsorption efficiency, thereby accelerating lipase-mediated hydrolysis through increased specific surface area [65]. (2) The higher encapsulation efficiency enabled enhanced gastric protection and controlled the intestinal release of bioactive compounds [66]. The plum seed AO was demonstrated to achieve 50% curcumin bioavailability, demonstrating comparable efficacy to advanced natural polymer-based delivery systems, including a walnut protein hydrolysate/naringenin emulsion (46% bioavailability) [67], chickpea protein isolate/pectin emulsion (51%) [53], and starch-based emulsion (51%) [68]. This performance parity with established encapsulation matrices confirmed that plum seed OBPs constitute a competitive natural alternative for bioactive compound delivery applications, combining equivalent functional performance with inherent biocompatibility advantages.

#### 3.3.7. Storage, Temperature, and Salt Stability

**Figure 19:**
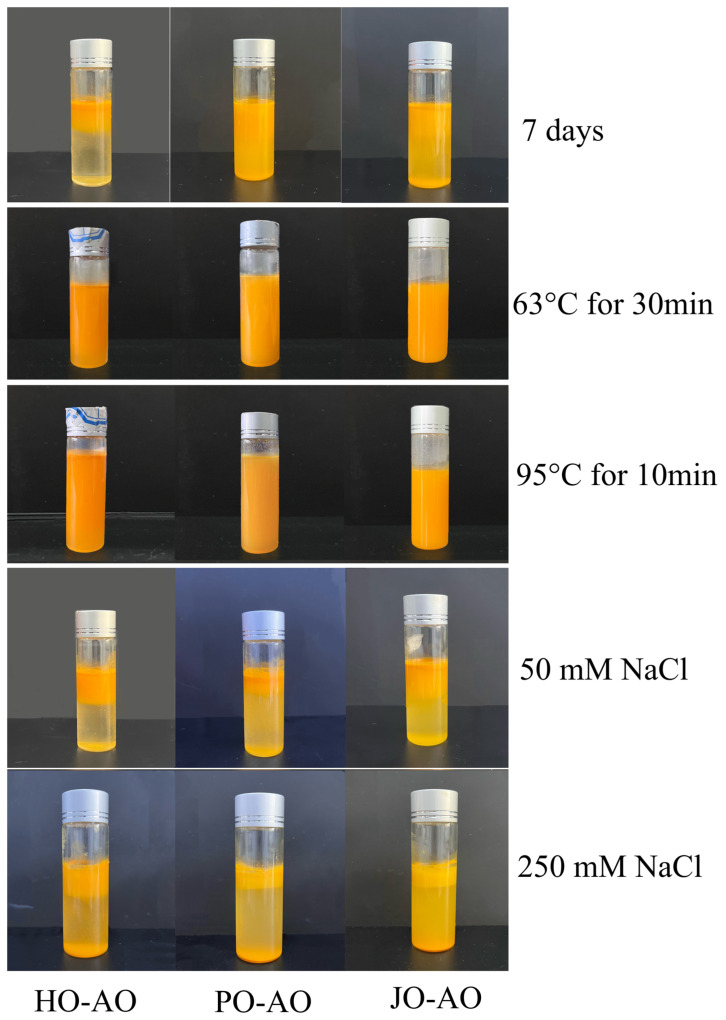
Effect of storage time, temperature, and NaCl concentration on the stability of artificial oleosomes (AOs) loaded with curcumin. HO-AO: artificial oleosomes stabilized by hemp seed oil body protein; PO-AO: artificial oleosomes stabilized by plum seed oil body protein (PO) and jujube seed oil body protein; JO-AO: artificial oleosomes stabilized by jujube seed oil body protein.

As demonstrated in Figure 19, the storage stability of AOs was systematically evaluated over a 7-day period at 4 °C. No visible serum layer was observed in plum seed OBP-based AOs after the storage duration, whereas significant phase separation was documented in hemp- and jujube-derived counterparts. This marked contrast in colloidal stability was mechanistically linked to two structural advantages of plum seed OBP-based AOs: (1) reduced particle dimensions that minimized gravitational separation forces, and (2) an elevated surface charge density that enhanced electrostatic stabilization through stronger interparticle repulsion [12]. Thermal stability assessments revealed that none of the AO variants exhibited macroscopic phase separation following thermal treatments, confirming their exceptional heat resistance. However, ionic strength challenges demonstrated critical stability limitations. Upon the addition of NaCl (50 or 250 mM), immediate destabilization was observed across all AOs. This salt-induced aggregation was mechanistically explained by charge screening effects, where Na^+^/Cl^−^ ions compressed the electrical double layer, effectively neutralizing the ζ-potential. The resultant reduction in electrostatic repulsion energy permitted dominant van der Waals attraction forces to drive irreversible flocculation [21].

## 4. Conclusions

This study provides critical insights into the structure–function relationships of seed-derived OBPs and their application in artificial oleosome fabrication. Key findings include the following: (1) SDS-PAGE and FTIR analyses revealed that plum seed OBPs possess higher random coil content (13%), facilitating enhanced surface hydrophobicity and smaller nanoaggregates; (2) plum seed OBPs exhibited superior emulsifying activity and stability, driven by synergistic effects of high surface charge, colloidal stability, and conformational flexibility; (3) AOs stabilized by plum seed OBPs achieved the highest encapsulation efficiency (92%) and bio-accessibility (50%), attributed to their small droplet size, strong electrostatic repulsion, and protective effects during simulated digestion. Furthermore, plum seed OBP-stabilized AOs demonstrated superior storage and thermal stability. These results position plum seed OBPs as a sustainable, high-performance alternative to synthetic emulsifiers in food and pharmaceutical applications. Future research should focus on scaling production and validating efficacy in in vivo models.

## Data Availability

All data generated or analyzed during this study are included in this manuscript.
